# Minimally Invasive Local Treatments for Bone and Pulmonary Metastases

**DOI:** 10.1155/2014/719394

**Published:** 2014-02-11

**Authors:** Meena Bedi, David M. King, Sean Tutton

**Affiliations:** ^1^Department of Radiation Oncology, Medical College of Wisconsin, 8701 Watertown Plank Road, Milwaukee, WI 53045, USA; ^2^Department of Orthopaedic Surgery, Medical College of Wisconsin, 8701 Watertown Plank Road, Milwaukee, WI 53045, USA; ^3^Department of Interventional Radiology, Medical College of Wisconsin, 8701 Watertown Plank Road, Milwaukee, WI 53045, USA

## Abstract

Surgery and chemotherapy have historically been the mainstay of treatment in patients with metastatic disease. However there are many alternative therapies available to relieve the symptoms and morbidity of metastases. In this paper, we review the role and highlight the advantages of minimally invasive techniques employed in patients with pulmonary and bone metastases.

## 1. Background

Multimodality therapy leads to excellent rates of local control in many malignancies. However, it is metastatic disease that usually dictates overall and disease-free survival in cancer patients. The most common sites of metastatic disease include the lung, liver, bone, and brain. Pain is the most common manifestation of osseous metastasis, whereas lung, liver, and brain metastases can lead to organ dysfunction.

Metastasis most commonly arises at the lung bases. Signs and symptoms of metastatic disease to the lung include, but are not limited to, cough, respiratory compromise, hemoptysis, dysphagia, and superior vena cava syndrome. Management is usually conducted with a palliative intent with standard treatment of chemotherapy. Although chemotherapy may lead to considerable response, side effects may be prominent and recurrence is common. Local therapy with surgery can lead to survival rates between 20 and 40% [1]. However, the number and location of metastases, as well as multiple comorbidities, make patients not always amenable to surgical resection.

Bone is the third most common site of metastasis and is a common cause of pain. Each year, it is estimated that over 100,000 patients will develop osseous metastasis, with prostate and breast cancer primaries accounting for 65–75% of these patients [2–4]. Although pain is the most common symptom of osseous metastasis, pathologic fractures secondary to cortical weakening of bone can also lead to increased morbidity with pain and dysfunction [5]. The most common location of pathologic fractures is the femur, followed by the humerus, acetabulum, tibia, and forearm [6].

Chemotherapy for painful bone metastases may be beneficial in widespread disease; however, pain response to this therapy is not well reported in the literature. Although local therapy with surgery repairs pathologic fractures and can lead to reduction of pain, improvement of function and quality of life, this management is typically not used solely for pain control.

Surgical intervention for both pulmonary and bone metastases can lead to complications such as pain, delays in wound healing, and infection. Thus, adjuvant treatment such as chemotherapy may be postponed. Minimally invasive techniques, alternatively, may be used for control of metastatic disease without the propensity for increasing complications.

The purpose of this paper is to describe the use of minimally invasive local therapies of radiation, radiopharmaceuticals, radiofrequency and cryoablation, and cementoplasty in the management of bone and pulmonary metastases.

## 2. Radiation Therapy

### 2.1. Local Field Radiation Therapy

Radiation therapy is oftentimes employed to palliate pain and other symptoms in patients with metastatic disease. Partial relief occurs in approximately 50% to 80% and complete pain relief occurs in approximately 30% to 50% of patients [7–10]. Several studies have attempted to determine the effectiveness of various dose and fractionation schemes, however, the optimal dose for pain control is not known.

RTOG 9714 was a phase III, prospective randomized control trial evaluating pain response in patients with 1 to 3 bony metastases in breast or prostate cancer [11]. Patients were randomized to a single fraction of radiation to 8 Gy versus 10 fractions of radiation to 30 Gy. Pain relief was assessed with the Brief Pain Inventory. There was no difference in the partial (50% versus 48%, resp.) and complete response (15% versus 18%, resp.). More patients required retreatment for their metastases in the single fraction arm, 18%, compared to the multi-fraction arm, 9% (*P* < 0.001). However, there was a significantly lower rate of grade-2-to-4 toxicity in the single fraction arm, 10% versus 17% (*P* = 0.002). There was no difference in late toxicities in either arm [11].

Three meta-analyses have also evaluated various fractionation schedules in patients with bony metastases [12–14]. Chow et al. reviewed 16 randomized trials, evaluating 5,000 patients, comparing radiation doses ranging from 8 Gy to 15 Gy delivered in a one fraction to 20 to 30 Gy over 3 to 10 fractions [12]. The primary outcomes examined were complete and overall response. Secondary outcomes assessed the rates of retreatment, pathological fracture, spinal cord compression, and acute toxicity [12].

Although response definitions, followup, and pain assessments varied between each study, there was no significant difference in overall response (58% versus 59%, resp.), complete response (23% versus 24%, resp.), or acute toxicity. However, there was a nonstatistically significant increase in risk of pathologic fractures and spinal cord compression in patients who underwent single fraction radiation compared to multifraction radiation. There was also an increase in the retreatment rate when radiation was delivered as a single fraction, 20%, compared to 8% when delivered over multiple fractions (*P* < 0.00001, 95% CI 1.76–3.56). The findings of comparable response rates, but higher retreatment rates, were also conferred in two other meta-analyses in patients with bony metastases [12–14].

### 2.2. Stereotactic Body Radiation Therapy

The development of stereotactic body radiotherapy (SBRT) originates from the use of stereotactic radiosurgery (SRS) in the treatment of CNS metastatic tumors, where a single fraction of high dose radiation using multiple beams precisely targets small intracranial tumors while minimizing radiation exposure to surrounding tissues. Due to the success in the treatment of CNS lesions, as well as the advancement in imaging, broader applications of radiosurgery have been developed to treat extracranial sites of disease.

SBRT employs conformal, high dose radiation delivery, over a limited number of fractions, for the treatment of small-to-moderate sized extracranial tumors. Advantages of SBRT include its unique radiobiological characteristics which lead to highly effective treatment of the target volume, while minimizing exposure to the surrounding tissue [15]. This is accomplished through the use of multiple beams, such that a small fraction of the total dose is administered through each beam, thereby effectively minimizing toxicity through the trajectory of the beam [15–18].

**Figure 1:**
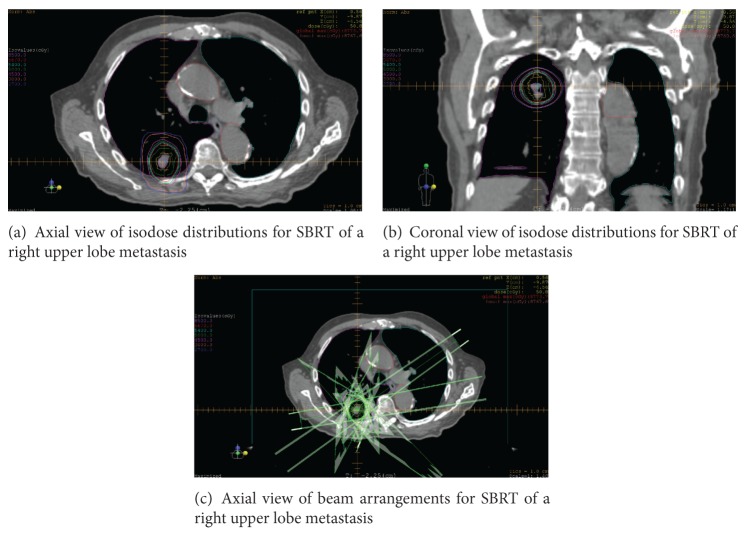
Axial view (a) and coronal view (b) of isodose distributions and beam arrangements (c) for SBRT of a right upper lobe metastasis.

Hypofractionated SBRT is an emerging method of treatment for metastatic disease in the lungs (Figures 1(a)–1(c)). Many studies have evaluated outcomes and toxicity in patients who have undergone SBRT for pulmonary oligometastasis from various tumor primaries [15]. Lesions were usually central or peripherally located with crude local control rates between 67 and 100% and 2-year survival ranging between 32 and 87% [16, 19–23]. Toxicity is acceptable with very few developing grade 3 or 4 complications (Table 1).

Ricardi et al. evaluated 61 patients with lung metastasis treated with SBRT. Doses ranged from 26 to 45 Gy in 1 to 4 fractions. With a median followup of 20.4 months, 2-year local control, overall survival, and progression free survival were 89%, 66.5%, and 32.4%, respectively. No patient had grade 4 toxicity, and only 1 patient had grade 3 toxicity [23].

Dhakal et al. assessed 52 patients with pulmonary sarcoma metastases. Fifteen patients were treated to 74 lesions using SBRT and compared to their non-SBRT cohort. The preferred treatment regimen was delivered over 2 weeks to 50 Gy in 5 fractions using conformal arcs or multiple coplanar beams. The 3-year local control in patients managed with SBRT was 82%. The median overall survival in patients treated with SBRT was 2.1 years versus 0.6 years in those who never received SBRT [21].

### 2.3. Radiopharmaceuticals

Bone-seeking radiopharmaceuticals are designed to selectively deliver radiation in osteoblastic metastases in hopes of improving pain control in those with multifocal disease. The uptake of radiotracers is dependent on calcification of normal tissue and the osteoblastic activity of the tumor. The discrepancy in bone turnover between normal and metastatic sites leads to improved integration of each radionuclide into metastatic bone. Thus targeted and focal radiation therapy can be simultaneously delivered to all sites in patients with widespread metastatic disease [24–28] (Table 2). A summary of the prospective studies done on systemic radionuclides commonly used in clinical practice is located in Table 3 [29–35].

Radionuclides are typically administered in an outpatient setting through intravenous (IV) access. Authorized administers inject the radiopharmaceutical over the course of approximately 1 to 2 minutes followed by a saline flush. After the IV has been removed, patients are provided with instructions for increased fluid intake and urinary excretion. Weekly blood counts are obtained to assess any change secondary to the therapy administered.

Phosphorous-32 (^32^P) was the first radionuclide to be consistently used in bone metastases and is available in an oral form, which allows for decreased cost and increased convenience. However, this radiotracer has fallen out of favor due to the high rates of myelotoxicity secondary to its longer range in targeted tissue and high energy decay [24–26, 28].

Strontium-89 (^89^Sr) is administered as an IV injection and is beta emitter with a half-life of 50.5 days. Because of chemical similarities with calcium, ^89^Sr is rapidly taken up in bony matrix, especially where active bone formation exists. ^89^Sr was one of the first radiopharmaceuticals approved for the treatment of widespread bone metastases; thus there is abundant data reporting on outcomes and pain response to this therapy. Overall pain response to ^89^Sr is approximately 60% to 90%, especially in patients with metastatic breast and prostate cancer [25, 36–38].

^89^Sr use has been studied alone and in conjunction with radiation and chemotherapy. Porter and McEwan prospectively evaluated 126 patients with hormone refractory prostate cancer that were randomized to radiation therapy followed by a single injection of ^89^Sr or radiation followed by placebo. Overall response rates were not significantly different in the two arms; however there was a decrease of the requirement for analgesics (2.4% versus 17.1%, *P* < 0.05) in favor of the combined modality group [31].

Samarium-152 ethylenediaminetetramethylenephosphonate (EDTMP) (^153^Sm-EDTMP) is a bone-seeking radioisotope with a short half-life of 46.3 hours that is slowly administered through IV injection. ^153^Sm is chelated to EDTMP to allow for delivery in areas of high bone turnover in patients with metastatic disease. Clinical response and experience with ^153^Sm is somewhat limited, but published reports have indicated pain response rates of approximately 70 to 80% [25, 26, 33–35].

Collins et al. evaluated 20 patients with escalated dose regimens of 1.0, 1.5, 2.0, 2.5, and 3.0 miCi/kg ^153^Sm EDTMP. The maximum tolerated dose was found to be 2.5 mCi/kg in this patient population. Overall pain relief occurred in 76% of patients within 1 to 2 weeks of administration [34].

Radium-223 (^223^Ra) is a radiopharmaceutical alpha-emitter with a half-life of 11.4 days that acts as a calcium analogue. ^223^Ra was recently approved in the use of hormone refractory metastatic prostate cancer [28]. The Alpharadin in Symptomatic Prostate Cancer Patients (ALSYMPCA) trial randomized 921 castrate resistant metastatic prostate cancer patients with 2 or more bone metastases to 6 injections of ^223^Ra or placebo. The primary endpoint was overall survival. In the updated analysis, median survival for patients who received ^223^Ra was 14.9 months compared to 11.3 months in the placebo group (*P* < 0.001). Time to increase in the first skeletal event (*P* < 0.001), time to increase in total alkaline phosphatase level (*P* < 0.001), and time to increase in PSA level (*P* < 0.001) were all improved with the use of ^223^Ra. There was no significant difference in grade-3-to-4 toxicity between the ^223^Ra and placebo groups [39].

Transient hematologic toxicity is the primary side effect of radiopharmaceuticals, especially thrombocytopenia and neutropenia. Grade-2-to-3 hematologic toxicity is not common and can occur in approximately 25% of patients. In approximately 10 to 20% of cases, a transient flare of bone pain occurs within 1 to 2 days. Less common side effects include loose stools, nausea and vomiting, hematuria, and heart palpitations [24–26].

Although conventional, stereotactic, and systemic radiation therapy may be used in the setting of metastatic disease, various histologies, such as renal cell carcinoma, are relatively radioresistant. As such, other minimally invasive methods may be used to improve local control and palliate symptoms.

## 3. Interventional Techniques

### 3.1. Radiofrequency Ablation

**Figure 2:**
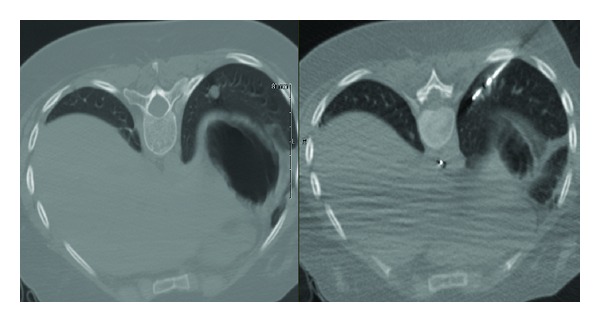
Treatment of a left lung sarcoma metastasis with radiofrequency ablation.

The susceptibility of malignant cells to extreme temperatures allows for the use of different techniques to treat metastatic disease. Radiofrequency ablation (RFA) employs temperatures as low as 41°C to cause tumor death [40, 41] and has been historically used in the treatment of unresectable tumors of the lung, liver, and kidney (Figure 2). This technique has been shown to provide excellent rates of local control and survival in patients with metastatic disease (Table 4) [42–46].

RFA is executed with the use of a percutaneously inserted electrode, typically under imaging guidance, which deposits energy in the form of an alternating electrical current to cause focal coagulation necrosis. Heat energy is distributed radially within the target tissue and a margin of normal tissue surrounding the tumor [47].

Yamakado et al. assessed 155 unresectable lung metastases from colorectal cancer in 71 patients treated with RFA. The 3-year overall survival was 46% and intrapulmonary recurrence occurred in 47% of patients in this cohort. Patients who had no extrapulmonary metastases and tumors ≤3 cm had a 3-year survival of 78%. On multivariate analysis, extrapulmonary metastasis (*P* < 0.02, CI 1.3–14.8) and tumor size >3 cm (*P* < 0.001, CI 3.4–52.6) lead to decreased survival. Pneumothorax, typically self-limited or requiring short term small bore chest tube, was the most common complication occurring in 37% of patients [42].

Nakamura et al. retrospectively reviewed 20 patients with 89 pulmonary metastases from sarcomas. The median followup was 18 months, in which the median survival was 12.9 months and the 3-year survival rate was 29%. The only prognostic indicator on univariate and multivariate analyses in this study was the ability to ablate all lung tumors. Patients with complete ablation of all tumors had a 1- and 3-year survival rate of 88.9% and 59.2%, respectively. Pneumothorax again was the most common complication, which occurred in 38% of patients. Thus, the authors concluded that RFA for pulmonary metastases was a safe and beneficial therapeutic option for appropriate candidates [43].

### 3.2. Cryoablation

**Figure 3:**
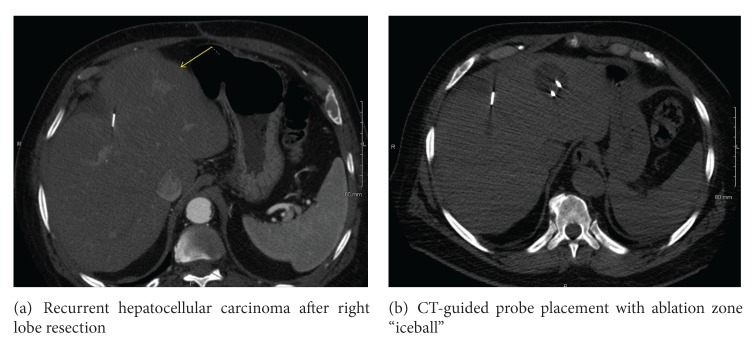
Recurrent hepatocellular carcinoma after right lobe resection (a) and ablation zone (b).

Whereas RFA applies heat to treat the targeted tissue, cryoablation exposes tumors to freezing temperatures to treat various malignancies. Cryoablation involves the insertion of dual chamber probe(s) into the target tissue. Typically, high pressure argon gas, which is supplied by a large in-room tank, is passed through the probe. Within a few seconds, there is rapid expansion and cooling, which leads to the production of temperatures of approximately −100°C. This generates a ball of ice up to 3.5 cm in size (Figures 3(a)-3(b)). Cell death is known to occur when temperatures are below −20°C. Multiple probes can be used to allow for the creation of larger balls of ice and, thus, the treatment of larger lesions [48].

Cell death from cryoablation is due to ice formation within the cell through immediate freezing of tissue adjacent to the probe. Gradual cooling away from the probe causes osmotic variation between the cell and membrane, leading to cell dehydration and eventual death [48].

Cryoablation has been utilized in the treatment of liver metastasis, particularly from colorectal primaries. Weaver et al. reviewed 136 patients with unresectable liver metastases from colorectal primaries who underwent 158 cryoablation procedures for tumor control. The median preoperative carcinoembryonic antigen (CEA) level was 14.4 ng/dL. Median survival was 30 months. Recurrent liver disease developed in 78% of patients, with 82% of these recurrences in the liver. Complication rates were comparable to liver resection and operative mortality was 3.7%. This led the authors to conclude that hepatic cryoablation is effective and safe in treating colorectal hepatic metastases under image guidance [49].

Cryoablation has also been used to palliate primary and metastatic bone lesions. Callstrom and colleagues prospectively assessed pain outcomes in 14 patients with osseous metastases from various tumors treated with cryoablation. Posttreatment scores for pain relief, worst pain, pain interference with daily activities, and narcotic medication use decreased with the use of cryoablation [50].

Advantages of cryoablation include the large ablation zone potential using multiple probes and ease of visualizing the “iceball” with CT guidance. Tuncali et al. reported complete and partial relief of pain in 6 of 19 and 11 of 19 patients with bone and soft tissue tumors, respectively, with a mean diameter of 5.2 cm [51].

### 3.3. Cementoplasty

Cementoplasty refers to the percutaneous injection of polymethylmethacrylate (PMMA) to mechanically stabilize the skeletal system and provide pain relief in patients with osteolytic bony metastases. This stabilization prevents further collapse and relieves pain by mitigating stress on each vertebral body treated. Cementoplasty includes procedures such as vertebroplasty, kyphoplasty, sacroplasty, and osteoplasty, and is typically performed by trained interventional radiologists and surgeons [48].

**Figure 4:**
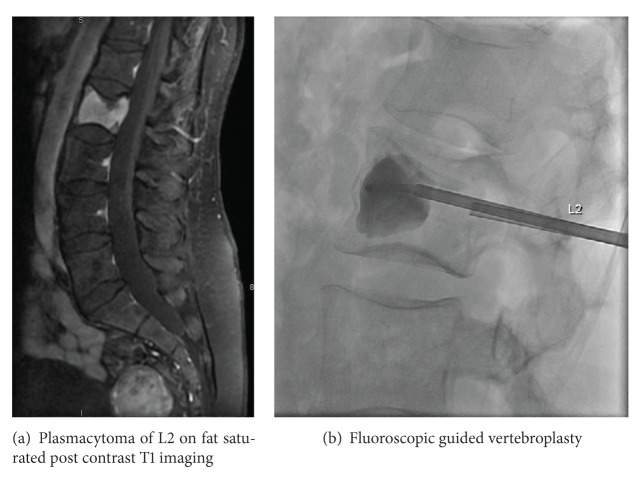
Plasmacytoma of L2 (a) treated with vertebroplasty (b).

The process of cementoplasty may be performed under general anesthesia or local anesthesia with conscious sedation or occasionally general anesthesia. A small incision is made, and, under image guidance with fluoroscopy, CT, or less commonly MRI, a trocar or needle is passed into the affected bone. Several commercially available cement preparations of PMMA, such as barium sulfate or tantalum, are mixed with materials to enhance radio-opacity, thereby allowing for better visualization and safer delivery with fluoroscopy. Evaluation of cement filling and potential leakage is also done through real-time imaging with fluoroscopy or CT-fluoroscopy (Figures 4(a)-4(b)). Adverse effects of the procedure itself include, but are not limited to, transient radicular pain, bleeding, infection, recurrent or adjacent level fracture, and rarely symptomatic pulmonary embolus [48]. Despite these risks, clinically significant complications remain very low in the literature.

Cementoplasty has been proven to be effective in pain relief in published reports [52–54]. Kelekis and colleagues reviewed 14 inoperable patients with painful bony metastases refractory to pain medications and radiation therapy. In this study, 23 lesions were treated with percutaneous cementoplasty using PMMA cement mixed with barium powder. All 14 patients had successful stabilization with cementoplasty and symptomatic pain relief was achieved within 24 hours after procedure in 13 of the 14 patients. Moreover, mobility after procedure was improved in 13 of the 14 cases by 1 week [52]. Many other studies have evaluated the success of cementoplasty alone or in combination with other interventional procedures (Table 5).

Hoffmann et al. reviewed 22 patients with 28 metastatic lesions in the spine, pelvis, and lower extremities treated with RA followed by cementoplasty. Pain relief occurred in all patients within 24 hours and after 3 months of the performed procedure. Moreover, the amount of pain medications used was also reduced in 15 of the 22 patients. The complication rates were also low [54].

## 4. Conclusion

Surgery and chemotherapy have long been the mainstay of treatment in metastatic disease. However, due to medical comorbidities, intolerance of systemic drug therapy, patient preference, and progression of disease, minimally invasive methods may be utilized in these scenarios. These techniques are becoming more applicable for the treatment of patients with metastatic disease and give the option of less invasive surgical approaches for palliation and local control. With the advancement of research and technology, new and innovative minimally invasive procedures are continually being developed and will benefit increasing numbers of patients with metastatic disease.

## Figures and Tables

**Table 1:** Summary of SBRT studies.

Author	Primary tumor	Number of patients	Median followup	Dose (median)	Outcomes	Toxicity
Le et al. [16]	Lung (91%), sarcoma (6%), and HCC (3%)	12	18 mo.	15–25 Gy in 1 fraction	LC at 15–20 Gy: 67% LC at 25 Gy: 56% 1 yr OS: 56%	19% pneumothorax (associated with fiducial placement)
Schefter et al. [19]	Colorectal (23.7%), sarcoma (18.4%), RCC (18.4%), lung (13.2%), and other (26.4%)	38	15.4 mo.	48–60 Gy in 3 fractions	1 yr LC: 100% 2 yr LC: 96% 2 yr OS: 39%	No ≥ G4 toxicity G3 toxicity: 8%
Wulf et al. [20]	Lung (45%), breast (10%), colon (8%), kidney (8%), sarcoma (8%), and other (18%)	25	14 mo.	26 Gy in 1 fraction	LC: 100% 1 yr OS: 68%	No ≥ G3 toxicity
Dhakal et al. [21]	Sarcoma (100%)	52	10.8 mo.	50 Gy in 5 fractions	2 yr LC: 88% 3 yr LC: 82% MS: 2/1 yrs	No ≥ G3 toxicity
Mehta et al. [22]	Sarcoma (100%)	16	20 mo.	54 Gy in 3-4 fractions	43 mo. LC: 94% 4 yr OS: 72%	No ≥ G2 toxicity
Ricardi et al. [23]	Lung (55.7%), colorectal (21.3%), pancreas (3.4%), HCC (3.4%), head and neck (3.4%), sarcoma (1.6%), and other (11.2%)	61	20.4 mo.	26 Gy in 1 fraction	2 yr LC: 89% 3 yr LC: 83.5% 2 yr OS: 66.5% 3 yr OS: 52.5%	G3 toxicity: 1.6%

**Table 2:** Characteristics of bone-seeking radiopharmaceuticals.

Radionuclide	Physical half-life	Energy max (MeV)	Decay	Mean range in tissue (mm)	Carrier
Phosphorous-32 (^32^P)	14.3 days	1.71	*β*	7.9	Orthophosphate
Strontium-89 (^89^P)	50.5 days	1.46	*β*	6.7	Chloride
Samarium-153 (^153^Sm)	46.3 hours	0.84	*β* and *γ*	3.4	EDTMP
Radium-223 (^223^Ra)	11.4 days	5.78	*α* and *γ*	<0.1	Chloride

**Table 3:** Summary of clinical trials evaluating radiopharmaceuticals.

Radionuclide	Cancer	Trial	Pain response	Survival
Phosphorous-32 (^32^P)				
Nair [29]	Breast, prostate, lung, and other	^ 32^P versus ^89^Sr	>50% reduction in pain in 93.3% of pts with ^89^Sr and 87.5% in ^32^P	Not reported
Strontium-89 (^89^Sr)				
Smeland et al. [30]	Prostate, breast, and other	RT + ^89^Sr versus RT + placebo	30% versus 20% (NS)	27 weeks versus 34 weeks (*P* = 0.6)
Porter and McEwan [31]	Prostate	RT + ^89^Sr versus RT + placebo	30–60% (complete response)	
Lewington et al. [32]	Prostate	^ 89^Sr versus placebo	Statistically significant decrease in pain	
Samarium-153 (^153^Sm)				
Serafini et al. [33]	Prostate, breast, lung, and other	^ 153^Sm versus placebo	72%	Not reported
Collins et al. [34]	Prostate	Phase I/II for ^153^Sm	76%	
Resche et al. [35]	Prostate, breast, lung, and other	^ 153^Sm 0.5 mCi/kg versus ^153^Sm 1.0 mCi/kg	55% versus 70% at week 4	
Radium-223 (^223^Ra)				
Parker et al. [39]	Prostate	^ 223^Ra versus placebo	Median time to first symptomatic skeletal event: 15.6 mo.	14.9 mo. versus 11.3 mo. (*P* < 0.001)

**Table 4:** Summary of RFA studies in metastatic disease.

Author	Primary tumor	Number of patients	Median followup	Outcomes	Most common toxicity
Yamakado et al. [42]	Colorectal	71	19 (mean)	3 yr OS: 46% Intrapulmonary recurrence: 47%	Pneumothorax (47%)
Nakamura et al. [43]	Sarcoma	20	18 mo.	1 yr OS: 88% 3 yr OS: 29% Incomplete ablation: 45%	Pneumothorax (65%)
Palussière et al. [44]	Sarcoma	29	50 mo.	1 yr OS: 92.2% 2 yr OS: 65.2% Incomplete ablation: 10%	Pneumothorax (67.8%)
Yan et al. [45]	Colorectal	55	24 mo.	1 yr OS: 85% 2 yr OS: 64% PFS: 15 mo.	Pneumothorax (29%)
King et al. [46]	Colorectal	19	24.3 mo.	1 yr LC: 90%	Pneumothorax (52%)

**Table 5:** Summary of cementoplasty studies in metastatic disease.

Author	*N*	Followup	Combination RFA	Location	Effective pain relief	Complication rate
Kelekis et al. [52]	14	9 mo. (mean)	No	Pubic rami and ischial tuberosities	92%	14.3%
Hierholzer et al. [53]	5	NR	No	Pelvis and femur	100%	0%
Hoffmann et al. [54]	25	7.7 (mean)	Yes	Spine, sacrum, acetabulum, and lower extremity	100%	0%
